# Delayed Diagnosis of Primary Pulmonary Sarcoma Arising from a Pulmonary Cyst in an Adult: A Case Report

**DOI:** 10.5761/atcs.cr.25-00097

**Published:** 2025-09-02

**Authors:** Yoshito Imamura, Taketo Kato, Satoko Shimada, Harushi Ueno, Shota Nakamura, Tetsuya Mizuno, Toyofumi Fengshi Chen-Yoshikawa

**Affiliations:** 1 Department of Thoracic Surgery, Nagoya University Graduate School of Medicine, Nagoya, Aichi, Japan; 2 Department of Pathology, Nagoya University Hospital, Nagoya, Aichi, Japan

**Keywords:** primary pulmonary sarcoma, congenital pulmonary airway malformation, pulmonary cystic diseases

## Abstract

Primary pulmonary sarcoma is a rare disease and is much less common than lung cancer among tumors arising from pulmonary cysts. We report the case of a female patient who showed multifocal cysts in the left S9–10. Follow-up computed tomography (CT) revealed that the cyst tended to regress, but the solid component of the cyst wall continued to thicken, growing to a 10-cm-diameter tumor. Thoracoscopic left lower lobectomy was performed to diagnose and treat the cystic lung lesions. For the pathology of the pulmonary cystic lesion, it was marked by solid tumors composed of proliferative atypical spindle cells, with some trapped bronchial tissue. Based on the imaging and pathological findings, the diagnosis was primary pulmonary sarcoma arising from the pulmonary cyst. The present case highlights that, even when a pulmonary cyst appears to shrink, careful follow-up and timely surgical consideration are warranted if cyst wall thickening is observed.

## Abbreviations

CPAM
: congenital pulmonary airway malformation
CT
: computed tomography
FDG-PET/CT
: ^18^F-fluorodeoxyglucose-positron emission tomography/computed tomography

## Introduction

Primary pulmonary sarcoma is a rare tumor accounting for 0.4%–1.1% of all lung neoplasms.^1)^ Among them, primary pulmonary sarcoma arising from a pulmonary cyst is commonly reported in children with congenital pulmonary airway malformation (CPAM).^2–5)^ However, most lung neoplasms arising from a pulmonary cyst are considered lung cancers in adults, and cases of sarcoma are very rare. Here, we report a case of a primary pulmonary sarcoma arising from a pulmonary cyst in an adult patient, with imaging findings showing transient shrinkage of the cyst before the definitive diagnosis.

## Case Presentation

**Fig. 1:**
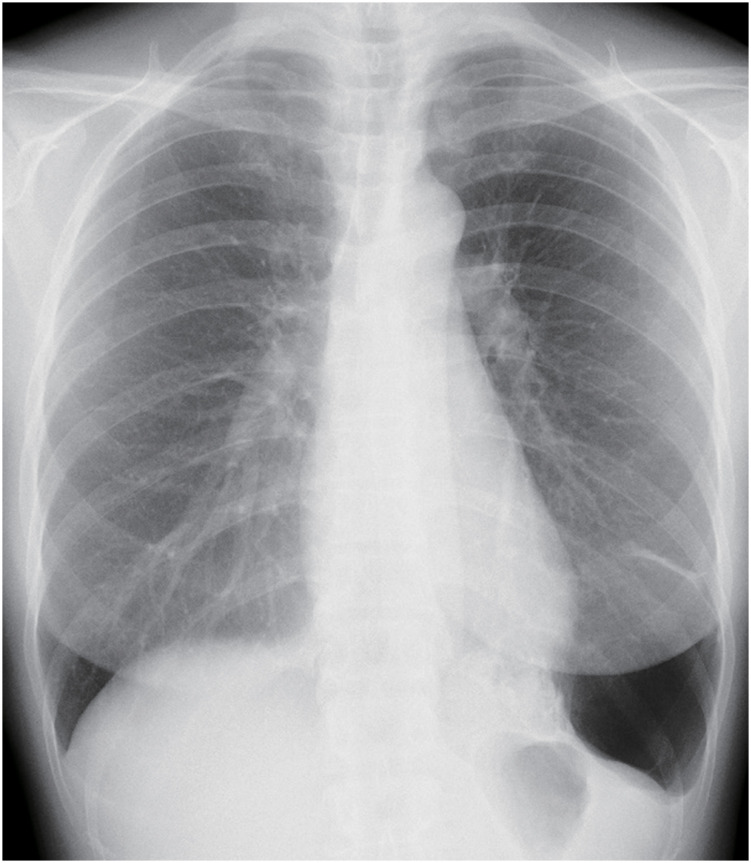
X-ray photograph taken during the initial health examination. Abnormalities with increased permeability were observed in the left lower lung field.

**Fig. 2:**
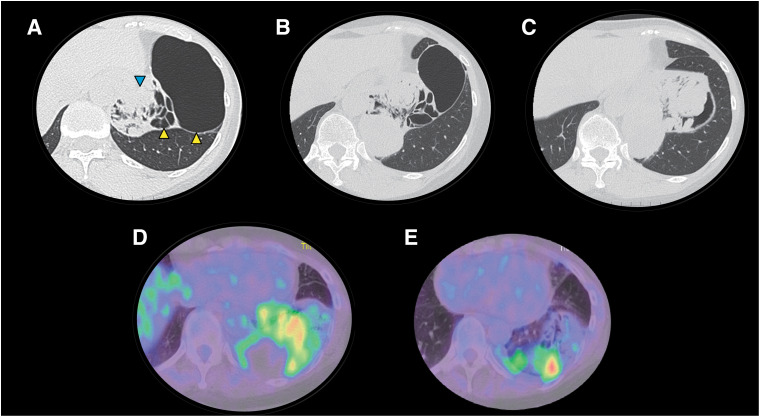
Radiological changes to the tumor and pulmonary cyst in the left lower lobe. (**A**) A chest CT scan at the previous hospital revealed a multifocal cyst (yellow arrowhead) and cyst wall nodule areas (blue arrowhead). (**B**) The cyst had shrunk 3 years after being detected. (**C**) The cyst had grown to a diameter of 10 cm with fluid effusion and progressive enlargement of the cyst wall 4 years after being detected. (**D**, **E**) FDG-PET/CT performed 5 years after detection revealed an SUVmax of 5.2 for the solid component of the cystic tumor, but slight accumulation in the fluid or cystic septum. CT: computed tomography; FDG-PET/CT: ^18^F-fluorodeoxyglucose-positron emission tomography/computed tomography; SUVmax: maximum standardized uptake value

**Fig. 3:**
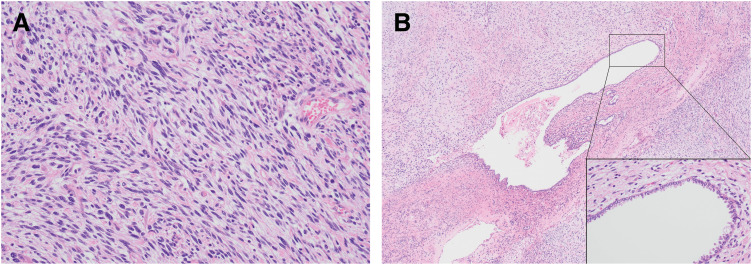
Histopathology of the primary pulmonary sarcoma located within the pulmonary cystic lesion. (**A**) The majority of the pulmonary cystic lesion was occupied by a solid tumor composed of proliferative atypical spindle cells (hematoxylin eosin stain ×200). (**B**) The pulmonary cystic lesion also contained several trapped cuboidal or pseudostratified cells, and ciliated columnar epithelium was also present (hematoxylin eosin stain ×40, ×200).

The patient was a woman in her 50s without any medical or family history and who did not smoke. A health examination revealed abnormalities in the left lower lung field, including increased permeability (**Fig. 1**). A chest computed tomography (CT) scan from the previous hospital revealed a left S9–10 multifocal cyst measured 11 cm and contained a 3-cm solid component in its wall (**Fig. 2A**). Given that there were no abnormal vessels from the aortic artery and no bronchioles surrounding the lesion, bronchial atresia was suspected, and the patient was observed. A CT scan performed at 3 years after the detection of the initial CT scan revealed that the cyst had shrunk to 9 cm, while the solid component had increased to 4 cm (**Fig. 2B**). Although the cyst was shrunk to 7 cm, the solid component of the cyst wall continued to thicken, growing to a 10-cm-diameter tumor with fluid effusion that appeared 4 years after the initial detection (**Fig. 2C**), prompting the referral to our facility. Our hospital’s ^18^F-fluorodeoxyglucose-positron emission tomography/CT (FDG-PET/CT), performed 5 years after detection, revealed a maximum standardized uptake value of 5.2 for the solid component of the cystic tumor. In contrast, slight accumulation was observed in the fluid or cystic septum (**Figs. 2D** and **2E**). Additionally, blood tests performed at our hospital revealed that the patient’s white blood cell count and C-reactive protein levels were within the normal ranges. A thoracoscopic lung resection was planned to diagnose and treat the cystic lung lesion. The tumor inside the cyst in the left lower lobe was attached to the mediastinal pleura near the pulmonary ligament, but it could be removed through the esophagus and inferior pulmonary vein. A partial lung resection was performed, and rapid intraoperative pathology confirmed the diagnosis of a pulmonary sarcoma; therefore, the tumor was completely resected via left lower lobectomy, and ND2a-1 lymph node dissection was performed. During surgery, the thickening of the pulmonary ligaments and pleura indicated chronic inflammation, and the pulmonary artery was dissected using a hilar approach without contact with the interlobar fissure due to the lung’s severely insufficient interlobar fissure. The histology of the pulmonary cyst revealed a solid tumor composed of proliferating atypical spindle cells (**Fig. 3A**), with no DICER1 mutation identified and with negative keratin staining. Furthermore, several trapped bronchial tissues composed of cuboidal or pseudostratified ciliated columnar epithelium were found, indicating a cystic background (**Fig. 3B**). Based on the radiological and pathological findings, the patient was diagnosed with a primary pulmonary sarcoma arising from the pulmonary cyst, pT4 (tumor size >7 cm) N0M0 Stage IIIA. The patient was discharged on postoperative day 8 without incident. She was followed up without adjuvant therapy and had no recurrence at 17 months postoperatively.

## Discussion

Primary pulmonary sarcoma is a rare type of malignant tumor originating in the lung.^1)^ Among such cases, those originating from pulmonary cysts are most frequently seen in children, commonly in those with CPAM.^2–5)^ Although there have been only a few reported cases of pulmonary sarcomas arising from pulmonary cysts in adults, the association of such cases with CPAM has not been reported (**Table 1**).^6–12)^ In previous cases, all patients were male, and only 2 patients were aged >50 years. Pneumothorax was observed in more than half of the cases, and the patients were relatively young, although asymptomatic cases took more than a year to be diagnosed, and there were no early deaths after surgery. Our patient had no obvious respiratory symptoms, and her condition was discovered at 54 years of age through a medical examination. Additionally, this is the first female case to be reported, which differs from the previous cases in terms of the background characteristics.

**Table 1:** Clinicopathological features of sarcoma arising from a pulmonary cyst in adults

Author, year	Age, gender	Symptoms	Size (cm), shape of cyst	Diagnostic period from detection of cysts	Therapy	Outcome (overall survival)
Iwasaki,^6)^ 1997	46, M	Asymptomatic	Unknown	Unknown	Lobectomy	Alive (7 years)
Miyazawa,^7)^ 2005	62, M	Asymptomatic	6.5, Unknown	10 years	Lobectomy	Alive (12 months)
Watzka,^8)^ 2009	41, M	Asymptomatic	4, Unifocal cyst	1 year 10 months	Segmentectomy	Alive (11 months)
Cummings,^9)^ 2010	21, M	Pneumothorax	1.5, Unifocal cyst	Unknown	Lobectomy	Alive (3 years)
Cummings,^9)^ 2010	25, M	Pneumothorax	Unknown	Unknown	Bullectomy	Unknown
Cummings,^9)^ 2010	29, M	Pneumothorax	Unknown	Unknown	Wedge resection + CT	Dead of disease (1 year 11 months)
Johnson,^10)^ 2017	25, M	Pneumothorax	1.1, Unknown	Unknown	Wedge resection + CT	Alive (2 years)
Guo,^11)^ 2017	37, M	Pneumothorax	Unknown	8 months	Lobectomy + CT	Dead of disease (3 months)
Rossi,^12)^ 2022	57, M	Pneumothorax	2, Unifocal cyst	Unknown	Wedge resection	Alive (4 months)
Present case	50, F	Asymptomatic	10, Multifocal cyst	5 years	Lobectomy	Alive (17 months)

CT: chemotherapy; F: female; M: male

Generally, pulmonary cysts can develop as a result of congenital anomalies or be acquired secondary to various diseases. Congenital pulmonary cystic diseases include CPAM, bronchogenic cysts, pulmonary sequestration, and bronchial atresia. Acquired pulmonary cysts may develop as a result of several factors, such as smoking, infections, and bronchial obstruction, but the present case was a never-smoker and did not show any clinical inflammatory symptoms causing acquired bronchial obstruction. In the case of synovial sarcoma, tumor tissue composed of epithelial-like and spindle-shaped cells indicates that cysts within the tumor are potentially derived from the sarcoma. However, in the present case, a diagnosis of synovial sarcoma was excluded, as the spindle cells in the tumor were negative for keratin stain. Additionally, given the minimal FDG accumulation in the cyst wall, the cyst was potentially a pre-existing structure infiltrated by tumor cells. Therefore, the present case indicates the possibility of a sarcoma associated with CPAM. CPAM is classified as a type 0–4 disease, and its diagnosis is based on pathological findings in pulmonary cysts composed of bronchial tissues.^3,13,14)^ Large cysts (>2 cm in size) form in CPAM type 1 and type 4 cases, whereas in other types, small cysts are produced. In the present case, the absence of a DICER1 mutation ruled out the presence of CPAM type 4. Our case partly met the diagnosis of CPAM type 1 based on the imaging findings (multiple pulmonary cysts suspected to be of congenital origin), and the pathologically trapped bronchial tissue indicated the presence of cystic lesions. However, a definitive diagnosis of CPAM type 1 could not be obtained because the cystic site was mostly occupied by a tumor at the time of surgery, which made pathological certification difficult. Reports of pulmonary sarcoma associated with CPAM are only found in children. Therefore, the present case may represent a rare condition implicating an association between pulmonary sarcoma and CPAM in adults.

Our paper is the first valuable report showing 5-year CT imaging changes of a primary pulmonary sarcoma arising from a pulmonary cyst, and the fact that the cyst shrank concurrently may have slowed the diagnosis. A previous study has reported a case of sarcoma in the lungs that retracted the surrounding pleura, changing the tumor’s shape and location.^15)^ Therefore, in the present case, it is possible that the tumor on the cyst wall expanded and pulled the cyst wall inwards, resulting in the collapse of the cystic septa and shrinkage of the cyst. Given that pulmonary cysts can potentially give rise to lung neoplasms, delayed treatment is unfavorable. Nonetheless, it is sometimes difficult to suspect the presence of a malignancy in the early stages, resulting in a delay in obtaining a definitive diagnosis.^13)^ In this case, surgery was considered when the patient was first referred to our hospital. However, short-term follow-up was initially selected, partly reflecting the patient’s preference. Even if the cyst is shrinking, careful follow-up and consideration of surgery are warranted in patients showing cyst wall thickening.

## Conclusions

We present a rare case of primary pulmonary sarcoma arising from a pulmonary cyst in an adult patient, with the cyst showing transient shrinkage before a definitive diagnosis was obtained. Careful follow-up and consideration of surgical intervention, with the risk of malignant complications, are necessary when cystic lung lesions demonstrate wall thickening.
